# Infectious Diseases Physician Management of Cryptococcal Meningitis in North America—Is Single High-Dose Liposomal Amphotericin B Being Used?

**DOI:** 10.1093/ofid/ofae120

**Published:** 2024-03-04

**Authors:** Nathan C Bahr, Susan E Beekmann, Philip M Polgreen, Jeremey B Walker, Andrej Spec, David R Boulware, John W Baddley

**Affiliations:** Division of Infectious Diseases, Department of Medicine, University of Kansas Medical Center, Kansas City, Kansas, USA; Division of Infectious Diseases and International Medicine, Department of Medicine, University of Minnesota, Minneapolis, Minnesota, USA; Division of Infectious Diseases, Department of Medicine, University of Iowa, Iowa City, Iowa, USA; Division of Infectious Diseases, Department of Medicine, University of Iowa, Iowa City, Iowa, USA; Division of Infectious Diseases, Department of Medicine, University of Alabama at Birmingham, Birmingham, Alabama, USA; Division of Infectious Diseases, Department of Medicine, Washington University in St. Louis, St. Louis, Missouri, USA; Division of Infectious Diseases and International Medicine, Department of Medicine, University of Minnesota, Minneapolis, Minnesota, USA; Division of Infectious Diseases, Department of Medicine, Johns Hopkins University School of Medicine, Baltimore, Maryland, USA

**Keywords:** cryptococcal meningitis, cryptococcosis, HIV/AIDS, organ transplantation

## Abstract

**Background:**

Several recent randomized trials have been conducted in resource-limited settings for cryptococcal meningitis that have rapidly innovated international guidelines. The 2010 Infectious Diseases Society of America (IDSA) cryptococcal meningitis guideline has not been updated with recent trials. The 2022 AMBITION-cm trial found that a single 10-mg/kg dose of liposomal amphotericin B plus daily flucytosine and fluconazole for 2 weeks was noninferior to 1 week of amphotericin B deoxycholate with flucytosine. It is unknown whether physicians in high-resource settings are using this regimen or more traditional regimens.

**Methods:**

We developed an electronic survey in June 2023 to better understand whether physician members of the IDSA Emerging Infections Network (EIN) and Mycoses Study Group Education and Research Consortium (MSG-ERC) had used the AMBITION-cm induction regimen, would use the regimen in hypothetical clinical scenarios, and what perceived barriers to use existed.

**Results:**

A total of 242 of 561 (43%) physicians responded to the survey, of whom 205 provided care for persons with cryptococcal meningitis in the last year. Overall, 29 (14%) had used the AMBITION-cm regimen, and 176 (86%) had not. In various hypothetical clinical scenarios, only ∼10% of 209 respondents selected the AMBITION-cm regimen as preferred. Perceived barriers to uptake included the applicability of trials performed in low-resource settings to high-resource settings, that the regimen is not recommended in the 2010 IDSA guidelines, and the applicability to persons without HIV.

**Conclusions:**

Most respondents had not used the single-dose liposomal amphotericin B regimen, but the regimen is being used. Further study of this regimen in other patient populations and settings is necessary.

The Infectious Diseases Society of America (IDSA) guideline for the management of cryptococcal disease was last updated in 2010 and recommends 2 weeks of amphotericin B deoxycholate (0.7–1.0 mg/kg daily) plus flucytosine (100 mg/kg daily) for the treatment of meningitis [1]. These recommendations for amphotericin deoxycholate combination therapy are based on a randomized controlled trial comparing amphotericin B deoxycholate with or without flucytosine [2]. Liposomal amphotericin B is mentioned as an option in persons with or predisposed to renal dysfunction based on a randomized trial of 267 HIV-positive persons that directly compared amphotericin B deoxycholate monotherapy at 0.7 mg/kg/d to liposomal amphotericin B monotherapy at either 3 mg/kg or 6 mg/kg [3, 4]. Liposomal amphotericin B at 3 mg/kg/d monotherapy did not meet a 10% noninferiority margin for 10-week survival when compared with amphotericin B deoxycholate [4]. The current 2023 US Health and Human Services HIV/AIDS Opportunistic Infection guidelines recommend liposomal amphotericin B 3–4 mg/kg/d with flucytosine for 2 weeks [5], although this regimen has not been studied in a randomized clinical trial.

High-quality randomized controlled trials in people with HIV have been completed since the publication of the most recent IDSA guidelines [6–8]. In 2018, the Antifungal Combinations for Treatment of Cryptococcal Meningitis in Africa (ACTA) trial was published [7]. This trial randomized 721 Africans with HIV-related cryptococcal meningitis to 1 of 5 regimens and found mortality to be lowest with 1 week of amphotericin B deoxycholate (1 mg/kg daily) plus flucytosine (100 mg/kg daily) followed by 1 week of fluconazole (1200 mg), before beginning consolidation therapy with fluconazole at 800 mg/d [7]. The World Health Organization (WHO) subsequently recommended this therapy as the preferred regimen for cryptococcal meningitis in persons with HIV [9].

Subsequently, Jarvis and colleagues published a trial investigating single-dose (10 mg/kg) liposomal amphotericin B treatment for cryptococcal meningitis, the AMBITION-cm trial [8]. This phase 3 trial randomly assigned 844 persons with HIV in 5 African countries to receive either (1) a single high dose of liposomal amphotericin B (10 mg/kg) on day 1 plus 14 days of flucytosine (100 mg/kg daily) plus fluconazole (1200 mg daily) OR (2) the WHO-recommended treatment (amphotericin B deoxycholate [1 mg/kg daily] plus flucytosine [100 mg/kg daily] for 7 days followed by 7 days of fluconazole [1200 mg daily]) for induction therapy [7–9]. The AMBITION-cm trial found the single high-dose liposomal amphotericin B–based regimen to be noninferior to the WHO-recommended regimen [8]. The AMBITION-cm regimen (a single high dose of liposomal amphotericin B [10 mg/kg] on day 1 plus 14 days of flucytosine [100 mg/kg daily] plus fluconazole [1200 mg daily]) is now recommended as the preferred regimen by the WHO for HIV-related cryptococcal meningitis [10].

The applicability of the AMBITION-cm regimen to high-income settings and in patients without HIV is not uniformly agreed upon. Although widespread use is advocated for by some based on its equivalent quantitative antifungal activity, lower cost, and lower toxicity, its uptake in the United States is unknown [11]. In the context of the AMBITION-cm trial's publication in March 2022 [8], we aimed to characterize preferred treatment regimens for cryptococcal meningitis in varied patient populations among practicing infectious diseases physicians in order to gain insight on perception and potential barriers to uptake.

## METHODS

We developed an electronic survey consisting of 7 questions and an additional space for free text commentary. Question 1 queried how many patients the respondent had seen with cryptococcal meningitis in the prior year. Only those people who saw 1 or more patients with cryptococcal meningitis were invited to continue the full survey. Of the 6 remaining questions, question 2 related to use of the AMBITION-cm regimen. Three questions presented hypothetical case-based scenarios and queried the respondent's preferred antifungal induction regimen. The scenarios focused on cryptococcal meningitis in an HIV-positive person, in liver transplantation, and in liver cirrhosis (ie, without HIV or other iatrogenic immunosuppression). Two questions sought to gain insight into why those who did not select the AMBITION-cm regimen in the case-based questions declined to do so for HIV or non-HIV scenarios. The full survey is available in the Supplementary Data. Table 1 contains the details of the possible induction antifungal regimens, provided as survey response options.

**Table 1.** Summary of Regimens Used in Hypothetical Cryptococcal Meningitis Scenarios

Induction Regimen	Amphotericin Component	Adjunctive Antifungals
AMBITION-cm trial [8]	Liposomal amphotericin B (10 mg/kg) once only	Flucytosine and fluconazole for 2 wk
IDSA [1, 5]^a^	Daily liposomal amphotericin B (3–4 mg/kg) for 2 wk	Flucytosine for 2 wk
ACTA trial [7]	Daily amphotericin B deoxycholate (1 mg/kg) for 1 wk	Flucytosine for 1 wk then fluconazole^b^ × 1 wk
Historical control [2]	Daily amphotericin B deoxycholate (0.7–1.0 mg/kg) for 2 wk	Flucytosine for 2 wk
IDSA (non-HIV, nontransplant) [1]	Daily amphotericin B deoxycholate (0.7–1.0 mg/kg) for ≥4 wk	Flucytosine for ≥4 wk
IDSA alternative (non-HIV, nontransplant) [1]	Daily liposomal amphotericin (3–4 mg/kg) for ≥4 wk	Flucytosine for ≥4 wk

Flucytosine is dosed at 100 mg/kg/d in ∼4 divided doses, and fluconazole is dosed at 1200 mg/d in ∼2 divided doses.

Abbreviations: ACTA, Antifungal Combinations for Treatment of Cryptococcal Meningitis in Africa; CDC, Centers for Disease Control and Prevention; HIVMA, Human Immunodeficiency Virus Medicine Association; IDSA, Infectious Diseases Society of America; NIH, National Institutes of Health.

^a^US NIH/CDC/HIVMA guidelines also recommend this regimen, but this was not part of the survey.

^b^Given after daily amphotericin B deoxycholate and flucytosine × 1 wk is completed.

The survey was sent to a subset of members of the IDSA Emerging Infections Network (EIN) who met the following eligibility criteria: (1) answered the previous EIN antifungal therapeutic drug monitoring survey [12], (2) reported on that survey that they provide care for patients with invasive fungal infections, (3) treat adults (not pediatric patients), and (4) are physicians. EIN is an IDSA and Centers for Disease Control and Prevention (CDC)–supported provider-based surveillance network [13]. EIN's membership makes up ∼20% of US Infectious Diseases (ID) physicians. The survey was also sent to members of the Mycoses Study Group Education and Research Consortium (MSG-ERG). The MSG-ERC is a nonprofit organization made up of physicians and scientists who are dedicated to providing continuing medical education and scientific/medical thought leadership related to invasive fungal infections [14]. The MSG-ERC was previously funded by the National Institute for Allergy and Infectious Diseases until 2007 [14]. The survey was sent to EIN members on June 28, 2023, with 2 reminders before survey closure on August 13, 2023. The survey was sent to MSG-ERG members with instructions not to complete if they had completed the survey through the EIN between August 1, 2023, and August 10, 2023.

The US Census Bureau geographic region [15] and years of ID experience were available for EIN members and MSG-ERC members. Practice setting was available for EIN members but not for MSG-ERC members. Statistical comparisons were not made between subgroups due to small numbers.

### Patient Consent

This study did not include factors necessitating patient consent and was institutional review board exempt.

## RESULTS

The survey was distributed to 361 EIN members who qualified under the eligibility criteria, of whom 222 (62%) responded. The survey was also distributed to 200 MSG-ERC members, of whom 20 (10%) responded. Overall, 242 (43%) of 561 responded to the survey. Of 242 responders, 37 (15%) stated that they did not see patients with cryptococcal meningitis and so opted out of the rest of the survey. There was a wide range of number of patients seen with cryptococcal meningitis, years of physician experience, and geographic diversity among the respondents (Table 2).

**Table 2.** Baseline Characteristics of Survey Respondents Overall, Those who Have and Have Not Used Single 10 mg/kg Dose Liposomal Amphotericin Induction Therapy for Cryptococcal Meningitis

Characteristic	Overall, No. (%)	Used AMBITION-cm Regimen (n = 29, 14%),^a^ No. (%)	Have Not Used AMBITION-cm Regimen (n = 176, 86%),^a^ No. (%)
Patients per year with cryptococcal meningitis	242 (100)		
None	37 (15)	NA	NA
<1	50 (21)	11 (22)	39 (78)
1–5	120 (50)	13 (11)	107 (89)
6–10	22 (9)	5 (23)	17 (77)
>10	13 (5)	0 (0)	13 (100)
Geographic region of practice^b^	205 (100)		
New England	10 (5)	1 (10)	9 (90)
Mid-Atlantic	22 (11)	3 (14)	19 (86)
East North Central	31 (15)	5 (16)	26 (84)
West North Central	29 (14)	10 (34)	19 (66)
South Atlantic	35 (17)	0 (0)	35 (100)
East South Central	7 (3)	0 (0)	7 (100)
West South Central	20 (10)	2 (10)	18 (90)
Mountain	6 (3)	2 (33)	4 (67)
Pacific	38 (19)	5 (13)	33 (87)
Canada and Puerto Rico	1 (0.5)	0 (0)	1 (100)
Other international	6 (3)	1 (17)	5 (83)
Experience since ID fellowship (n = 222)			
<5 y	36 (16)	6 (17)	30 (83)
5–14 y	76 (34)	8/67 (12)	59/67 (88)
15–24 y	44 (20)	5/43 (12)	38/43 (88)
≥25 y	66 (30)	9/57 (16)	48/57 (84)
Primary clinical setting (n = 185)			
City/county hospital	6 (3)	0 (0)	6 (100)
Community hospital	42 (23)	4 (10)	38 (90)
Non-university teaching hospital	56 (30)	6 (11)	50 (89)
University hospital	73 (39)	13 (18)	60 (82)
VA hospital	8 (4)	1 (13)	7 (88)

AMBITION-cm regimen: A single high dose of liposomal amphotericin B (10 mg/kg) on day 1 plus 14 d of flucytosine (100 mg/kg daily) plus fluconazole (1200 mg daily).

Abbreviation: NA, not applicable.

^a^Includes only those respondents who stated that they see patients with cryptococcal meningitis (n = 205 for patients seen per year and geographic location, n = 203 for experience since ID fellowship, n = 185 for primary clinical setting). Some percentages do not add up to 100% due to rounding. Not all respondents answered all questions.

^b^States in the US Census Bureau divisions: New England (Maine, New Hampshire, Vermont, Massachusetts, Rhode Island, Connecticut); Mid-Atlantic (New York, New Jersey, Pennsylvania); East North Central (Ohio, Indiana, Illinois, Michigan, Wisconsin); West North Central (Minnesota, Iowa, Missouri, North Dakota, South Dakota, Nebraska, Kansas); South Atlantic (Delaware, Maryland, District of Columbia, Virginia, West Virginia, North Carolina, South Carolina, Georgia, Florida); East South Central (Kentucky, Tennessee, Alabama, Mississippi); West South Central (Arkansas, Louisiana, Oklahoma, Texas); Mountain (Montana, Idaho, Wyoming, Colorado, New Mexico, Arizona, Utah, Nevada); Pacific (Washington, Oregon, California, Alaska, Hawaii).

Of 205 respondents who reported seeing patients with cryptococcal meningitis, 29 (14%) stated that they had used the AMBITION-cm induction regimen, and 176 (86%) had not. Of those 176 who had not used the regimen, 28 (16%) stated that they were not aware of the regimen, and 148 (84%) were aware of the regimen but had chosen to not use it. Of those 29 who stated that they had used the regimen, 13 (45%) used the regimen in advanced HIV only, 5 (17%) in patients without HIV but with other immune-compromising conditions, and 11 (38%) had used it in both persons with HIV and those without HIV.

**Figure 1.**
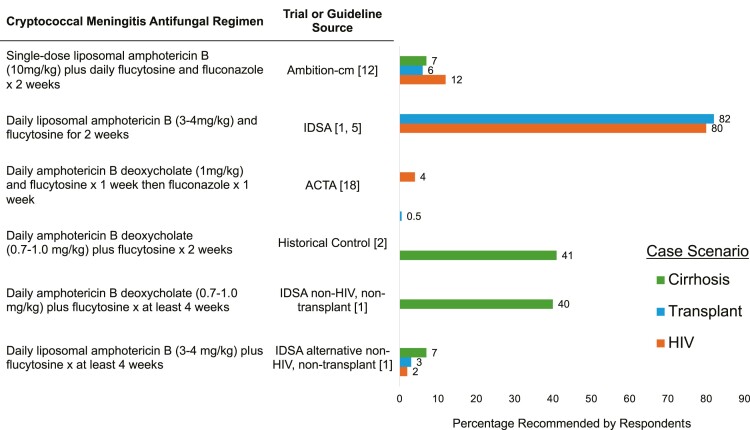
Responses to hypothetical clinical scenarios for patients with cryptococcal meningitis and underlying HIV, liver transplant, or liver cirrhosis. The x-axis is the percentage of respondents. Flucytosine was dosed at 100 mg/kg daily, and fluconazole was dosed at 1200 mg daily. Other regimen refers to other preferred regimens selected by the respondents. Respondents who selected that they did not see the patient type (HIV n = 3 [1%], transplant n = 15 [7%], cirrhosis n = 8 [3.5%]) are not included. Abbreviation: IDSA, Infectious Diseases Society of America.

In assessing hypothetical clinical scenarios, respondents offered their preferred induction regimen to treat patients with cryptococcal meningitis with different underlying conditions: (1) HIV, (2) liver transplant, or (3) liver cirrhosis. Each question gave the option to choose a regimen not listed with a space for comment; these comments are available in the Supplementary Data. Figure 1 shows responses for each clinical scenario.

In a patient with advanced HIV, 25 (12%) respondents stated that they would use the AMBITION-CM regimen, 165 (80%) stated that they would use the 2-week IDSA regimen, 8 (4%) stated that they would use the 1-week amphotericin-5FC regimen, 4 (2%) stated that they would use another regimen, and 3 (1%) stated that they do not care for this patient population. In a patient who received a liver transplant 2 years prior who was on tacrolimus and mycophenolate mofetil, 13 (6%) said that they would use the AMBITION-cm regimen, 169 (82%) said that they would use the IDSA regimen, 1 (0.5%) said that they would use the historical control regimen, 7 (3%) said that they would use another regimen, and 15 (7%) said that they did not treat this patient population. In a patient with liver cirrhosis without a history of HIV or transplantation, 15 (7%) would use the AMBITION-cm regimen, 85 (41%) would use the IDSA nontransplant, would use the non-HIV regimen, 83 (40%) would use the IDSA alternative nontransplant, non-HIV regimen, 14 (7%) would use another regimen, and 1 (0.5%) stated that they did not treat this patient population.

Respondents were asked their reason(s) for not choosing the AMBITION-cm regimen in the patient with HIV and could select multiple responses. Fifteen respondents (7%) did not answer, and 22 (11%) selected not applicable as they used the AMBITION-cm regimen, leaving 168 respondents. Thirty-three (20%) selected that they had not heard of the AMBITION-cm study and would need to review the data, 54 (32%) selected that nobody they know was using the regimen and/or they were not comfortable with the regimen, 19 (11%) said that the data are not convincing/issues with study design and/or how the study was conducted, 73 (43%) selected that they did not believe the study applied to a high-resource setting as it was conducted in a low-resource setting, and 81 (48%) selected that US guidelines did not recommend the regimen.

For non-HIV patients, 10 respondents (5%) did not answer, and 13 (6%) stated that this was not applicable as they would use the AMBITION-cm regimen, leaving 182 respondents. Thirty-two (18%) had not heard of the regimen and would need to review the data, 48 (26%) said that nobody they know is using the regimen/uncomfortable with the regimen, 14 (8%) said that the data are not convincing/issue with study design or conduct, 51 (28%) selected that the study was done in a low-resource setting and did not apply to a high-resource setting, 85 (47%) selected that this study was done among people with HIV and did not apply to those without HIV, and 73 (40%) selected that US guidelines did not endorse the regimen.

An additional field was available for overall comments, which are available in the Supplementary Data. Selected representative comments include: “Would like to see updated consensus guidance from IDSA on management of cryptococcal meningitis (overdue!)”; “AMBITION compared single high dose LAmB [liposomal amphotericin B] to AmBd [amphotericin deoxycholate B]. I would use single high dose LAmB in a patient (HIV or non-HIV) who wants to leave the hospital against medical advice or for whom the risk of adverse effects of amphotericin would lead to unacceptable morbidity. I would love to see a future trial comparing high dose LAmB to IDSA recommended 2 week LAmB induction”; and “Would love to have more data in transplant recipients, and/or have the AST/IDSA come out with formal/updated guidelines.”

## DISCUSSION

In this survey of infectious disease physicians who care for patients with cryptococcal meningitis, the use of the WHO-recommended AMBITION-cm single-dose liposomal amphotericin B regimen was low, with only 13% of respondents stating that they had used the regimen in the first year since publication [8]. When presented with hypothetical patient scenarios, more respondents favored giving the AMBITION-cm regimen in a patient with advanced HIV, when compared with other non-HIV patient populations, although 6%–7% of respondents selected the AMBITION-cm regimen for non-HIV patients as well. In the hypothetical scenarios, most respondents selected the regimens recommended in the 2010 IDSA guideline. The overall response rate was 43%, but it is likely that this was actually higher given that many MSG-ERC members were likely queried in the initial EIN survey and so did not respond to the MSG-ERG iteration of the survey.

The most common reasons for not using the AMBITION-cm regimen in the hypothetical patient with HIV were that the regimen is not recommended in the IDSA guidelines and that the study was performed in a low-resource setting. The most common reasons for not using the AMBITION-cm regimen in the non-HIV patient scenarios were that the study was performed in people with HIV and that it was not recommended by IDSA guidelines, although other common reasons included the study being conducted in a low-resource setting and being uncomfortable with the regimen/no colleagues using the regimen.

This survey gives helpful insight into treatment decisions for a subset of ID physicians practicing primarily in the United States [11]. There are some physicians utilizing the AMBITION-cm regimen, but use is not widespread. One could infer that those using the regimen may concur with the recent publication from Harrison and colleagues regarding the toxicity, cost and efficacy of the AMBITION-cm regimen in high-income settings. Additionally, one could also infer that many physicians would like to see a study similar to AMBITION-cm take place in a high-resource setting and/or in non-HIV populations. This would be a major undertaking and require many more centers than were involved in the AMBITION-cm trial or the other major HIV-associated cryptococcal meningitis trials that have been conducted in low-resource settings over the past decade, given the lower disease incidence in higher-resource settings generally. In addition, it would be a costly study and take many years to complete. In the absence of similar high-quality clinical trials in other settings or populations, utilizing creative internet-based clinical trial approaches might be helpful, as would data reporting from real-world practices and analysis of observational data capturing variations in treatment. In particular, comparative studies for those treated with the AMBITION-cm regimen or other regimens in high-income settings and/or non-HIV populations would be useful. We now know that some US physicians are using the regimen, so outcomes regarding these patients are of interest.

There was minimal variation regarding whether physicians used the AMBITION-cm regimen by patients with cryptococcal meningitis seen per year, geographic region, years in practice since fellowship, or practice setting.

It is important to regularly update influential guidelines like the IDSA guidelines. With the last update having been in 2010, significant data are not accounted for in the current IDSA guidelines for cryptococcal meningitis. Updating IDSA and other guidelines is not a small task. Formally and systematically reviewing the data in an unbiased way using primarily the time of volunteers requires significant commitment. Yet, our results support that guidelines influence physician decision-making. Whether the AMBITION-cm regimen is recommended by updated IDSA guidelines, its review and consideration by a guidelines panel would be helpful for many physicians. Despite 2021 US NIH/CDC/HIVMA guidelines and 2022 WHO guidelines having been published and other guidelines soon to be published, US ID physicians clearly incorporate IDSA guidelines routinely into clinical practice. Further, evidence-based interventions are estimated to take 17 years to reach clinical practice [16]. We would expect that even with universal acceptance of the AMBITION-cm trial's applicability to high-income settings (which we did not find here), wholescale adoption would not be rapid. One possible limitation of the study is self-selection bias either because the group that responded to the survey was largely made up of those who responded to a previous survey about antifungal therapeutic drug monitoring or because persons with strong feelings about whether to use the AMBITION-cm regimen would be more likely to respond to the survey. It is also possible that the framing of the study question may have led to biases. For instance, labeling the regimen in the IDSA guidelines as the IDSA regimen may have reminded respondents of the society’s recommendations and influenced their answers. It is also possible that the respondents were influenced by social desirability bias to give answers that they thought the authors or their peers would prefer, although the survey was confidential. Lastly, though overall the sample was robust, some subanalyses had small numbers and should be interpreted cautiously.

In conclusion, a minority of EIN and MSG/ERC member ID physicians are using the AMBITION-cm single-dose liposomal amphotericin B regimen for cryptococcal meningitis induction. Physicians commonly cite that the trial was done in a low-resource setting and is not recommended by IDSA guidelines as barriers to use. In persons without HIV, the study having been conducted in people with HIV is also seen as a barrier. Further study of the regimen in additional populations/settings would likely be of interest to the ID community, but additional large clinical trials may not be feasible in all populations.

## Supplementary Material

ofae120_Supplementary_Data

## Supplementary Data

Supplementary materials are available at *Open Forum Infectious Diseases* online. Consisting of data provided by the authors to benefit the reader, the posted materials are not copyedited and are the sole responsibility of the authors, so questions or comments should be addressed to the corresponding author.
