# Supplementary material for: Infectious Diseases Physician Management of Cryptococcal Meningitis in North America—Is Single High-Dose Liposomal Amphotericin B Being Used?
Source: Open Forum Infect Dis. 2024 Mar 4;11(6):ofae120. doi: 10.1093/ofid/ofae120 (PMC11181173; doi:10.1093/ofid/ofae120)
Supplement: ofae120_Supplementary_Data [file ofae120_supplementary_data.zip › Supplement 1_survey.docx]

**
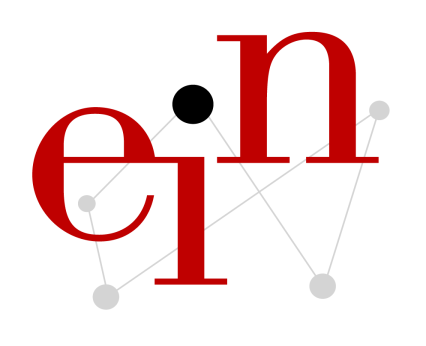
**

**INFECTIOUS DISEASES SOCIETY OF AMERICA**

**EMERGING INFECTIONS NETWORK QUERY:**

**Management of Cryptococcal Meningitis**

**by Infectious Disease Physicians**

IDSA guidelines for cryptococcal meningitis were last updated in 2010 (*Clinical Infectious Diseases*, Volume 50, Issue 3, 1 February 2010, Pages 291–322) and the federal HIV.gov guidelines for cryptococcosis were last updated in July 2021 (<https://clinicalinfo.hiv.gov/en/guidelines/hiv-clinical-guidelines-adult-and-adolescent-opportunistic-infections/cryptococcosis?view=full>). The ACTA trial also informs guidelines on treatment of cryptococcal meningitis (<https://www.nejm.org/doi/pdf/10.1056/NEJMoa1710922>). In 2022, publication of the AMBITION-cm trial data from Africa provided new evidence related to the management of cryptococcal meningitis (Jarvis JN et al, NEJM 2022). The World Health Organization guidelines (<https://www.who.int/publications/i/item/9789240052178>) now recommend the AMBITION-cm regimen for HIV-associated cryptococcal meningitis. We are interested in how ID physicians in the U.S. are currently treating cryptococcal meningitis.

**The purpose of this survey is to understand current practice patterns for the management of cryptococcal meningitis in North America.**

Definitions

AmB = amphotericin B deoxycholate

5FC = flucytosine

**EMERGING INFECTIONS NETWORK QUERY**

**Management of Cryptococcal Meningitis**

Name:

**1. How many patients do you see with cryptococcal meningitis per year on average?**

__ <1 (not every year) __ 1-5 __ 6-10 __ >10

**__** I do not see patients with cryptococcal meningitis **--** STOP HERE and submit

**2. Have you used a single, high dose, liposomal amphotericin-based (AMBITION-cm) regimen for cryptococcal meningitis?**

__ No, not aware of this regimen

__ No, do not use this regimen

__ Yes, in patients with advanced HIV

__ Yes, in HIV-negative patients with other immunocompromising conditions

__ Yes, in both patients with and without advanced HIV

For the following 3 patients who present with cryptococcal meningitis, please indicate the induction regimen you would choose for each:

**3. A 50-yo male with HIV (normal kidney function) and a CD4 count of 5 cells/mcL:**

__ Single dose liposomal amphotericin (10mg/kg) on Day 1 + 5FC 100mg/kg/day x2 weeks + fluconazole 1200mg daily x2 weeks (AMBITION-cm regimen)

__ Liposomal amphotericin 3-4mg/kg/day + 5FC 100mg/kg/day x 2 weeks (IDSA regimen)

__ AmB 1mg/kg/day + 5FC 100mg/kg/day for 1 week followed by fluconazole 1200mg daily for 1 week (ACTA regimen)

__ Other regimen, *specify:*

__ N/A, do not care for patients like this

**4. A 50-yo female (HIV-negative) with a history of liver transplant 2 years prior on tacrolimus + mycophenolate mofetil:**

__ Single dose liposomal amphotericin (10mg/kg) on Day 1 + 5FC 100mg/kg/day x2 weeks + fluconazole 1200mg daily x2 weeks (AMBITION -cm regimen)

__ Liposomal amphotericin 3-4mg/kg/day + 5FC 100mg/kg/day x 2 weeks (IDSA regimen)

__ AmB 0.7-1.0 mg/kg/day + 5FC 100mg/kg/day x 2 weeks (historical control regimen)

__ Other regimen, *specify:*

__ N/A, do not care for patients like this

**5. A 43-yo male with cirrhosis (HIV-negative):**

__ Single dose liposomal amphotericin (10mg/kg) on Day 1 + 5FC 100mg/kg/day x2 weeks + fluconazole 1200mg daily x2 weeks (AMBITION -cm regimen)

__ AmB 0.7-1.0 mg/kg/day + 5FC 100mg/kg/day x at least 4 weeks (IDSA regimen)

__ Liposomal amphotericin 3-4mg/kg/day + 5-FC 100mg/kg/day x at least 4 weeks (IDSA alternative regimen)

__ Other regimen, *specify:*

__ N/A, do not care for patients like this

**6. If you did not use the AMBITION-cm trial regimen** (the first answer option for each scenario above) **for HIV patients,** **please select any reason/barrier below that applies:**

__ N/A, use the AMBITION-cm trial regimen

__ I hadn’t heard of this study and would need to review the data before considering

__ Nobody who I know is using this regimen, and/or I’m not comfortable with the regimen

__ The data are not convincing to me; I have issues with study design and/or how it was conducted

__ The study was done in a low-resource setting; I don’t think that it applies to high-resource settings

__ U.S. guidelines do not recommend this regimen

**7. If you did not use the AMBITION-cm trial regimen** (the first answer option for each scenario above) **for non-HIV patients,** **please select any reason/barrier below that applies:**

__ N/A, use the AMBITION-cm trial regimen

__ I hadn’t heard of this study and would need to review the data before considering

__ Nobody who I know is using this regimen, and/or I’m not comfortable with the regimen

__ The data are not convincing to me; I have issues with study design and/or how it was conducted

__ The study was done in a low-resource setting; I don’t think that it applies to high-resource settings

__ The study was done in people with HIV, so I am unsure if it applies to patients without HIV

__ U.S. guidelines do not recommend this regimen

**8. Any final comments about treatment of cryptococcal meningitis?**

*Thank you for completing this survey!*
