# Supplementary material for: Infectious Diseases Physician Management of Cryptococcal Meningitis in North America—Is Single High-Dose Liposomal Amphotericin B Being Used?
Source: Open Forum Infect Dis. 2024 Mar 4;11(6):ofae120. doi: 10.1093/ofid/ofae120 (PMC11181173; doi:10.1093/ofid/ofae120)
Supplement: ofae120_Supplementary_Data [file ofae120_supplementary_data.zip › Supplment 2_ final data report.docx]

**
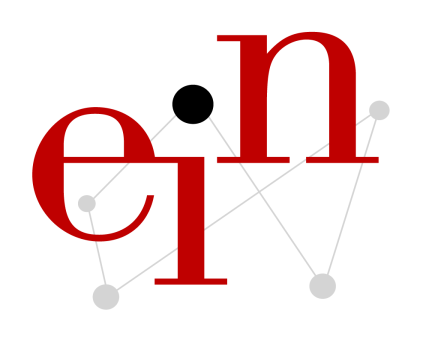
**

**Infectious Diseases Society of America**

**Emerging Infections Network**

**Report for Query:**

**‘Management of Cryptococcal Meningitis by Infectious Disease Physicians and**

**by MSGERC Clinicians’**

**This survey link was sent to a subset of EIN members who met the following criteria: 1) answered the antifungal therapeutic drug monitoring survey, 2) reported on that survey that they provide care for patients with invasive fungal infections, 3) treat adults (not pediatric patients), and 4) are physicians (not other healthcare professionals).**

**Three emailed requests to answer this query were sent to the subset of 361 EIN physician members -- on 6/28/23, 7/25/23 and 8/8/23.**

222 of 361 (62%) EIN members responded from 6/28 through 8/13/23.

**This survey link was sent to Mycoses Study Group Education & Research Consortium clinician members on 8/1/23.**

20 MSGERC members responded from 8/1 through 8/10/23.

*Note: Not all respondents answered all questions, so totals for individual questions vary.*

**Question 1. How many patients do you see with cryptococcal meningitis per year on average?**

Do not see patients with cryptococcal meningitis - OPT OUT of survey 37 (15%)

< 1 (not every year) 50 (21%)

1-5 120 (50%)

6-10 22 (9%)

>10 13 (5%)

The 37 respondents who opted out stopped here.

**The 205 respondents who see patients with cryptococcal meningitis answered at least some of the remaining questions.**

**Practice characteristics of the 205 EIN member respondents**:

Region: New England 10 (5%)

Mid Atlantic 22 (11%)

East North Central 31 (15%)

West North Central 29 (14%)

South Atlantic 35 (17%)

East South Central 7 (3%)

West South Central 20 (10%)

Mountain 6 (3%)

Pacific 38 (19%)

Canada and Puerto Rico 1 (0.5%)

Other international 6 (3%)

Years’ experience since ID fellowship: <5 years 36 (16%)

5-14 76 (34%)

15-24 44 (20%)

≥25 66 (30%)

**Question 2. Have you used a single, high dose, liposomal amphotericin-based (AMBITION-cm) regimen for cryptococcal meningitis?**

No, not aware of this regimen 28 (14%)

No, do not use this regimen 148 (72%)

Yes, in patients with advanced HIV 13 (6%)

Yes, in HIV-negative patients w/ other immunocompromising conditions 5 (2%)

Yes, in both patients with and without advanced HIV 11 (5%)

For the following 3 patients who present with cryptococcal meningitis, please indicate the induction regimen you would choose for each:

**Question 3. A 50-yo male with HIV (normal kidney function) and a CD4 count of 5 cells/mcL:**

| Single dose liposomal amphotericin (10mg/kg) on Day 1 + 5FC 100mg/kg/day x2 weeks + fluconazole 1200mg daily x2 weeks (**AMBITION-cm regimen**) | 25 (12%) |
| --- | --- |
| Liposomal amphotericin 3-4mg/kg/day + 5FC 100mg/kg/day x 2 weeks (**IDSA regimen**) | 165 (80%) |
| AmB 1mg/kg/day + 5FC 100mg/kg/day for 1 week followed by fluconazole 1200mg daily for 1 week (**ACTA regimen**) | 8 (4%) |
| Other regimen* | 4 (2%) |
| N/A, do not care for patients like this | 3 (1%) |

**Other, specified by 6 respondents*: “Do both AMBITION and IDSA regimens depending upon clinical and social situation”; “Lip ampho 5 mg/kg/day”; “Need more data”; Liposomal amphotericin 5 mg/kg/day+ 5FC 100 mg/kg/day x 2 weeks”; “Would use the AMBITION-cm regimen in a relatively uncomplicated CM case (ie. not a case of recurrent CM after failing or being poorly adherent to prior regimen)”; “IDSA regimen without 5FC because we don't have it in Mexico”

**Question 4. A 50-yo female (HIV-negative) with a history of liver transplant 2 years prior on tacrolimus + mycophenolate mofetil:**

| Single dose liposomal amphotericin (10mg/kg) on Day 1 + 5FC 100mg/kg/day x2 weeks + fluconazole 1200mg daily x2 weeks (**AMBITION-cm regimen**) | 13 (6%) |
| --- | --- |
| Liposomal amphotericin 3-4mg/kg/day + 5FC 100mg/kg/day x 2 weeks (**IDSA regimen**) | 169 (82%) |
| AmB 0.7-1.0 mg/kg/day + 5FC 100mg/kg/day x 2 weeks (**historical control regimen**) | 1 (0.5%) |
| Other regimen* | 7 (3%) |
| N/A, do not care for patients like this | 15 (7%) |

**Other, specified by 8 respondents*: “AmBisome 5 mg/kg”; “Lamb + maybe 5FC if counts ok then azole depends on many factors”; “Liposomal ampho + 5FC for at least 2 weeks pending culture clearance/clinical improvement”; “Need more data”; “Would typically use (3) but I do feel comfortable with the single dose regimen”; “Liposomal amphotericin 5 mg/kg/day + 5FC 100mg/kg/day x 3-4 weeks”; “AMBITION cm regimen without 5FC because we don't have in Mexico”; “IDSA regimen but will tailor duration of induction regimen to organism identity ( gatti vs neoformans) and clearance CSF culture”

**Question 5. A 43-yo male with cirrhosis (HIV-negative):**

| Single dose liposomal amphotericin (10mg/kg) on Day 1 + 5FC 100mg/kg/day x2 weeks + fluconazole 1200mg daily x2 weeks (**AMBITION-cm regimen**) | 15 (7%) |
| --- | --- |
| AmB 0.7-1.0 mg/kg/day + 5FC 100mg/kg/day x at least 4 weeks (**IDSA regimen**) | 85 (41%) |
| Liposomal amphotericin 3-4mg/kg/day + 5-FC 100mg/kg/day x at least 4 weeks (**IDSA alternative regimen**) | 83 (40%) |
| Other regimen* | 14 (7%) |
| N/A, do not care for patients like this | 7 (3%) |
| *[Not answered]* | 1 (0.5%) |

**Other, specified by 14 respondents*: “AmBisome 5 mg/kg”; “Depending on the severity of infection and status of their cirrhosis”; “LAMB x7-10 (doubt cirrhotics would tolerate if hypersplenism ) then azole”; “Liposomal AmB 3-4 mg/kg + 5-FC 100 mg/kg/day minimum 2 weeks with reassessment (LP, clinical response, renal tolerance of AmB) and decide next steps”; “Liposomal amphotericin 3-4mg/kg/day + 5FC 100mg/kg/day x 2 weeks”; “Need more data”; “Same as IDSA regimen for transplant”; “Same as previous two patients with close LFT monitoring”; “Liposomal amphotericin 3-4mg/kg/day + 5-FC 100mg/kg/day x at least 2 weeks”; “Liposomal amphotericin 5 mg/kg/day + 5FC 100mg/kg/day x 3-4 weeks”; “I would base my decision on the CSF CrAg titer, OP, and renal function. I would probably lean towards the 4 weeks ambisome regimen (especially if I am concerned of high fungal burden and ICP) unless renal function cannot tolerate and I need to think of an alternative regimen. More data needed.”; “IDSA alternative regimen without 5FC because we don't have it in Mexico”; “IDSA regimen but will tailor duration of induction regimen to organism identity (gatti vs neoformans) and clearance CSF culture”; “Liposomal amphotericin 3-4 mg/kg/day + F-FC 100mg/kg/day x 2 weeks”

**Question 6. If you did not use the AMBITION-cm trial regimen** (the first answer option for each scenario above) **for HIV patients,** **please select any reason/barrier below that applies:**

*[Select all that apply; numbers add to >100%]*

| N/A, use the AMBITION-cm trial regimen | 22 (11%) |
| --- | --- |
| I hadn’t heard of this study and would need to review the data before considering | 33 (16%) |
| Nobody who I know is using this regimen, and/or I’m not comfortable with the regimen | 54 (26%) |
| The data are not convincing to me; I have issues with study design and/or how it was conducted | 19 (9%) |
| The study was done in a low-resource setting; I don’t think that it applies to high-resource settings | 73 (36%) |
| U.S. guidelines do not recommend this regimen | 81 (40%) |
| *[Not answered]* | 15 (7%) |

**Question 7. If you did not use the AMBITION-cm trial regimen** (the first answer option for each scenario above) **for non-HIV patients,** **please select any reason/barrier below that applies:**

*[Select all that apply; numbers add to >100%]*

| N/A, use the AMBITION-cm trial regimen | 13 (6%) |
| --- | --- |
| I hadn’t heard of this study and would need to review the data before considering | 32 (16%) |
| Nobody who I know is using this regimen, and/or I’m not comfortable with the regimen | 48 (23%) |
| The data are not convincing to me; I have issues with study design and/or how it was conducted | 14 (7%) |
| The study was done in a low-resource setting; I don’t think that it applies to high-resource settings | 51 (25%) |
| The study was done in people with HIV, so I am unsure if it applies to patients without HIV | 85 (41%) |
| U.S. guidelines do not recommend this regimen | 73 (36%) |
| *[Not answered]* | 10 (5%) |

**Question 8. Any final comments about treatment of cryptococcal meningitis:**

*[open text field**; 44 respondents added text]*

Excel file with open-text field answers may be viewed here: <http://www.int-med.uiowa.edu/Research/EIN/CryptoMening_Comments.pdf>
